# Phonological analysis of substitution errors of patients with apraxia of speech

**DOI:** 10.1590/S1980-57642010DN40100010

**Published:** 2010

**Authors:** Maysa Luchesi Cera, Karin Zazo Ortiz

**Affiliations:** I Speech Therapist, Specialization in Human Communication Disorders at the Federal University of São Paulo (UNIFESP), Reading for Masters in Human Communication Disorders (UNIFESP).; 2 Speech Therapist, PhD, Professor of the Department of Speech Therapy of the Federal University of São Paulo.

**Keywords:** articulation disorders, apraxias, diagnosis, rehabilitation of speech and language disorders

## Abstract

The literature on apraxia of speech describes the types and characteristics of phonological errors in this disorder. In general, phonemes affected by errors are described, but the distinctive features involved have not yet been investigated.

**Objective:**

To analyze the features involved in substitution errors produced by Brazilian-Portuguese speakers with apraxia of speech.

**Methods:**

20 adults with apraxia of speech were assessed. Phonological analysis of the distinctive features involved in substitution type errors was carried out using the protocol for the evaluation of verbal and non-verbal apraxia.

**Results:**

The most affected features were: voiced, continuant, high, anterior, coronal, posterior. Moreover, the mean of the substitutions of marked to markedness features was statistically greater than the markedness to marked features.

**Conclusions:**

This study contributes toward a better characterization of the phonological errors found in apraxia of speech, thereby helping to diagnose communication disorders and the selection criteria of phonemes for rehabilitation in these patients.

## Abstract (pt)

A literatura sobre a apraxia de fala descreve os tipos e as características fonológicas dos erros neste distúrbio. Em geral, são descritos os fonemas acometidos pelos erros, porém os traços distintivos envolvidos ainda não foram estudados.

**Objetivo:**

Analisar os traços fonológicos envolvidos nas substituições cometidas por apráxicos falantes do português, falado no Brasil.

**Métodos:**

Participaram do estudo 20 adultos com apraxia de fala. Foi realizada análise fonológica dos traços distintivos envolvidos nos erros do tipo substituição cometidos na aplicação do protocolo de avaliação da apraxia verbal e não-verbal.

**Resultados:**

Os traços mais acometidos foram, respectivamente: sonoro, contínuo, alto, anterior, coronal, posterior. Além disso, as médias das substituições do traço marcado para o não marcado foram estatisticamente maiores do que do traço não marcado para o marcado.

**Conclusões:**

Este estudo contribui para uma melhor caracterização dos erros fonológicos da apraxia de fala, auxilia no diagnóstico dos distúrbios da comunicação, bem como nos critérios de seleção dos fonemas para a reabilitação destes quadros.

Apraxia of speech is an articulation disorder resulting from impairment, secondary to brain damage, of the capacity to program the positioning of speech musculature and the sequencing of muscle movements for volitional production of phonemes.^1^

Studies^2-21^ have interpreted the manifestations of individuals with apraxia of speech as an impairment of linguistic phonological processing, motor impairment, or both. The notion of apraxia of speech as a motor disorder is now generally accepted. However, it frequently co-occurs with aphasia and differentiating between the respective motor and linguistic impairments has proven difficult.

The study by Dogil and Mayer (1998) proposed a view of apraxia of speech based on a linguistic theory. Considering that pure apraxia of speech affects only verbal performance, and patients with apraxia of speech produce not only phonetic errors (e.g. distortions) but also phonemic errors (e.g. substitutions, deletions, insertions, reduplications, metatheses, etc.), it is reasonable to consider a linguistic interpretation of this language disturbance.^2^

However, some studies have revealed differences in the terminology adopted to describe the distortion.^3-5^ Odell^3^ described 14 types of distortion, and observed prolongation as the most common, followed by devoicing. Cera and Ortiz.^4^ did not considered prolongation as a distortion and devoicing was considered a substitution type error since one phoneme is replaced by another; Johns and Darley^5^ defined distortion as the inaccurate production of a phoneme which is consequently rendered unrecognizable. Besides the distortion, other types of errors are often described in apraxia of speech.

Darley et al.^6^ showed that the most common errors among apraxics were: substitutions, additions, repetitions and phonemic prolongations. Peach and Tonkovich^7^ observed substitution errors, followed by addition, repetition, intrusion, omission and other error types.

Studies^1-13^ in the literature describe the types of error found in apraxia of speech, the results of which show errors of substitution to be the most frequent error type presented by this patient group.^4-7.10,15-18^ A previous Brazilian study revealed that the most frequently affected phonemes in Brazilian speakers with apraxia of speech (/β/, /λ/ and /ʒ/) were different to those typically reported in studies involving other languages, and suggested that the errors might be influenced by phonological rules of the language.^19^

Cera and Ortiz^19^ described the most frequently affected phonemes in Brazilian speakers with apraxia of speech. The sample obtained in the is previous study was examined in the present study analyzing the distinctive features involved in substitution errors.

Analysis of the distinctive features involved in errors of speech by apraxics provides a deeper understanding of this disorder and can improve the management and treatment of these patients.

Therefore, the aim of the present study was to analyze the distinctive features involved in substitutions committed by Brazilian Portuguese-speaking apraxics.

## Methods

The final sample comprised 20 adults aged between 41 and 80 years, with 11 men and 9 women, assessed at the Center for Speech and Hearing Investigation in Neuropsycholinguistics of Unifesp, who were diagnosed with apraxia of speech during 2007, according to the presence of the following types of error: metathesis, anticipation, reiteration, substitution, repetition, omission, addition, self-correction, trial-and-error, where these errors are typical of the oral production of apraxics.

For study inclusion, subjects had to present a neurological diagnosis of a single lesion to the left-hemisphere and be speakers of Brazilian Portuguese. The sample also included apraxics with associated aphasia since few patients presented solely apraxia of speech.

Individuals who presented marked expression deficit, characterized by suppression or severely reduced oral capacity; impaired auditory comprehension preventing task execution; clinical history or diagnosis of previous neurological conditions (such as epilepsy, head trauma with loss of consciousness of longer than 15 minutes); uncorrected hearing or visual disturbances; history of severe depression or psychiatric disorders; and use of psychotropic drugs, were excluded.

Speech assessment was carried out using the verbal praxic component of the protocol for evaluation of verbal and non-verbal apraxia,^20^ which entails tests of word and sentence repetition, automatic and spontaneous speech and oral reading aloud. The "Cookie Theft" test from the Boston Diagnostic Aphasia Examination was used to elicit spontaneous speech production.^22^ Patient speech was digitally recorded using a SONY MP3 player and concomitantly transcribed.

A phonological analysis of the features involved in substitutions was carried out, focusing solely on substitutions occurring in consonant segments. All substitution errors that occurred across all tasks were analyzed. The frequency of this type of error was small in vowels, and so these substitutions were not analyzed. This analysis was performed based on the distinctive features model proposed by Chomsky and Halle (1968) (apud Yavas, Hernandorena and Lamprecht, 2001)^23^ and used the Consonant Segments Matrix for the Portuguese language. The features contained in this matrix include: sonorant, syllabic, consonantal, continuant, strident, delayed release, nasal, lateral, anterior, coronal, high, low, posterior and voiced. The involvement of each feature in every substitution was registered from more to less, when the substitution occurred from marked to markedness features, and from the less to the more, where markedness features were substituted by marked features. Features were assessed based on the occurrence of each substitution and by recording the features involved in the substitution for each occurrence.

This study was approved by the Research Ethics Committee of the Federal University of Sao Paulo (UNIFESP) under protocol number 1105/07. All participants signed a free and informed consent form.

Descriptive statistical analysis was carried out on the data gathered. Differences among means for continuous data were analyzed using Wilcoxon's test. A probability (p) value of less than 0.05 was considered statistically significant and all tests were two-tailed. Ninety five percent confidence intervals (CI) were calculated for differences between means.

## Results

Three patients were diagnosed with hemorrhagic cerebral stroke while the remainder had suffered ischemic strokes. All apraxic patients bar one, were also aphasics: ten patients had mixed aphasia, four had Broca’s aphasia, one had conduction aphasia, one had transcortical sensory type aphasia, one had anomic aphasia and two had minor language impairments.

The sites of lesions were confirmed through a neurological assessment and according to imaging exams. Six subjects presented brain lesion in the left temporoparietal region, 4 in the left fronto-temporal region, 3 in the left fronto-temporal region, 2 in the left parietal region and 2 presented lesions in left frontal region, 1 in the left temporal region, 1 fronto-temporo-parietal region and 1 in the left parietal-occipital region.

The distribution of the number of features involved in substitutions is depicted in Table 1.

**Table 1:** Distribution of the number of distinctive features involved in substitutions

Feature	- → +	+ → -	Total
Voiced	4	94	98
Continuant	31	54	85
High	32	51	83
Anterior	48	31	79
Coronal	34	34	68
Posterior	12	28	40
Lateral	8	17	25
Consonantal	0	18	18
Sonorant	5	7	12
Strident	3	8	11
Nasal	4	6	10
Delayed release	0	0	0
Syllabic	0	0	0

+, marked features; -, markedness features.

The mean of marked (more) to markedness (less) features was statistically greater than the markedness to marked features (26.7±26.9versus 14.0±16.3; Z= –2.22; p=0.026).

## Discussion

Table 1 shows the distribution of distinctive features involved in the substitutions. Previous studies that have phonologically characterized errors in apraxia of speech identified the phonemes affected but did not explore the distinctive features involved in the errors.^3,5.7-8.11,21^ Therefore, we shall discuss our findings by drawing on the results of studies which have described the phonemes most frequently produced erroneously by apraxics. We found only one study which investigated the features involved in the emissive errors committed by speakers of Portuguese. The sample in question however, comprised only aphasics.^24^ In our study, we found the most affected features to be: voiced, continuant, high, low, anterior, coronal and posterior. By contrast, the previous study involving phonological analysis of commutation and permutation committed in repetition tests by aphasic subjects, revealed the features in which errors most frequently occurred to be as follows: coronal, continuant, anterior, strident, posterior, high and voiced. The present study found the same features to be most frequently involved in phonological errors, suggesting that this error type is directly influenced by the Portuguese language. However, the frequency of occurrence and their respective order differed between the two studies. This may be due to the difference in subjects’ diagnoses in the studies, since Parente^24^ assessed aphasics while we studied apraxics, although all but one of our subjects presented an associated aphasic diagnoses. Other methodological differences were also present between the studies. For instance, the cited study included only six subjects thereby limiting the statistical analyses of the findings, and subjects were assessed using repetition of real and nonwords, while types of errors analyzed included commutation (substitutions) and permutation (omissions, additions and reversals).

The voiced feature was the most frequently affected by the substitution error. Regarding this aspect, we found a study in the literature which showed that consonant distortion surpassed all other errors, including sound substitution, and identified 14 types of distortion (prolongation, followed by devoicing).^3^ Devoicing was one of the most frequent errors in the cited study, a finding in line with our results, since the voicing feature was the most frequently affected by praxic errors, even though we considered devoicing as a substitution error because the marked phoneme was substituted by a markedness phoneme. Similarly, a Brazilian study which assessed the speech of apraxics also observed a high rate of substitution of voiced by voiceless phonemes.^19^

In another Brazilian study, in which phonological analysis was performed in children diagnosed with phonological disorders, Wertzner et al.^25^ identified that phonological processes produced by the majority of the subjects were unvoicing of plosives and fricatives, the most commonly occurring in the groups with phonological disorder.^25^ The feature involved in unvoicing is the voiced feature, and was therefore the most affected feature in the study, mirroring our results. The study in question involved a very different population to our study, given that it included children with phonological disorder, whereas adults with apraxia of speech were assessed in our study. Although these authors assessed a specific population, the similarity in findings leads us to believe that the production of a voiced phoneme is more complex. Nevertheless, we should emphasize that the substitutions seen in the speech of the two populations may have different underlying causes, given that the children with phonological disorders presented changes in phonological acquisition of sounds of speech. Nevertheless, we may hypothesize that the difficulties encountered by both groups involve a praxic emissive component, as well as specific interference from the language itself.

Moreover, it was noted that the mean substitution of the marked to markedness feature was statistically higher than the reverse. This finding was also observed in the study by Blumstein,^26^ in which the spontaneous emission of 200 words by Broca, conduction and Wernicke aphasics were analyzed.^26^ In addition, the literature reports that the number of errors increases with complexity of motor adjustment needed for articulation,^8,10-12^ where motor adjustment is more complex in the production of marked features than markedness phonemes. Therefore, the results of our study suggest that the complexity of the stimulus to be emitted influences the occurrence of the error, such that marked phonemes are more susceptible to errors. However, this finding is not in agreement with the results of the study by Wolk,^21^ who analyzed the production of consonants in three aphasics with apraxia of speech and reported that low complexity phonemes (markedness) tended to be substituted by high complexity phonemes (marked).^21^ This discrepancy can be explained by the difference in the languages of the patients assessed, as well as by the small sample analyzed in the study by Wolk.^21^ This aspect was not analyzed in recent studies.

Thus, we observed that voiced phonemes are more susceptible to error, as are phonemes with marked features, and that the distinctive features involved in substitutions seem to be influenced by language. Thus, the present study contributes by better characterizing the phonological errors found in apraxia of speech, thereby helping to diagnose communication disorders. Additionally, our findings may improve the phoneme selection criteria for rehabilitation in these patients, particularly with regard to the voiced feature and marked phonemes, which should not be elected early in therapy, where the more easily-produced voiceless and markedness phonemes should be used instead.
